# Associations of socioeconomic determinants with community clinic awareness and visitation among women: evidence from Bangladesh Demographic and Health Survey-2011

**DOI:** 10.1186/s13104-015-1374-7

**Published:** 2015-10-21

**Authors:** Mohammad Abul Bashar Sarker, Md. Harun-Or-Rashid, Joshua A. Reyer, Tomoya Hirosawa, Yoshitoku Yoshida, Mohammod Monirul Islam, Md. Ruhul Furkan Siddique, Shaila Hossain, Junichi Sakamoto, Nobuyuki Hamajima

**Affiliations:** Department of Healthcare Administration, Nagoya University Graduate School of Medicine, 65, Tsurumai-Cho, Showa-ku, Nagoya, 466-8550 Japan; National Control Laboratory, Institute of Public Health (IPH), Dhaka, Bangladesh; Department of Rehabilitation, Yonaha General Hospital, Mie, Japan; Directorate General of Health Services, Dhaka, Bangladesh; National Institute of Preventive and Social Medicine (NIPSOM), Dhaka, Bangladesh; Tokai Central Hospital, Tokai, Japan

**Keywords:** Bangladesh’s women, Community clinic, Demographic and Health Survey, Primary health care

## Abstract

**Background:**

Although Bangladesh has achieved tremendous success in health care over the last four decades, it still lagged behind in the areas of maternal and child malnutrition and primary health care (PHC). To increase access to PHC, the Bangladesh government established approximately 18,000 community clinics (CCs). The purpose of this study was to examine the associations of socioeconomic determinants of women aged 12–49 years with the CCs awareness and visitation.

**Methods:**

We analyzed secondary data provided by Bangladesh Demographic and Health Survey-2011. A two-stage cluster sampling was used to collect the data. A total of 18,222 ever married women aged 12–49 years were identified from selected households and 17,842 were interviewed. The main outcome measures of our study were awareness and visitation of CCs. Bivariate logistic regression was used to calculate odds ratio (OR) and 95 % confidence interval (CI) to examine the associations between the awareness and visiting CCs with socioeconomic determinants.

**Results:**

Low prevalence of awareness about CC (18 %) was observed among studied women and only 17 % of them visited CCs. Significant associations (P < 0.05) with CCs awareness and visitation were observed among aged 20–29 years (adjusted OR = 1.18; 95 % CI = 1.03–1.35 and adjusted OR = 1.49; 95 % CI = 1.05–2.11), primary education (adjusted OR = 1.20; 95 % CI = 1.08–1.34 and adjusted OR = 1.37; 95 % CI = 1.05–1.78), and poorest family (adjusted OR = 1.21; 95 % CI = 1.03–1.42 and adjusted OR = 2.36; 95 % CI = 1.56–3.55, respectively), after controlling potential confounders.

**Conclusions:**

Awareness and visitation of CCs were found to be positively associated with lower economic conditions, young age, and primary education. Awareness and access to CCs might be increased through community activities that involve health care workers. The government should also lower barriers to PHC access through CCs by providing adequate logistics, such as human resources and equipment.

## Background

Bangladesh achieved tremendous success in health care over the last four decades, despite many challenges such as gender equity, women’s education, natural disasters, low national GDP, and high poverty [1]. The country was recognized as having “good health at low cost” due to its huge improvement in child and maternal mortality [2]. Although the United Nations recognized Bangladesh for its exemplary progress towards Millennium Development Goal (MDG) 4 in child mortality and for being on right track to achieve MDG 5 in maternal mortality, it still lagged behind in the area of maternal and child malnutrition and primary health care (PHC). From 1991 to 2010, maternal mortality ratios and infant mortality rates declined from 574 to 194 per 100,000 live births and 96 to 39 per 1000 live births respectively [1, 3]. The lowest skilled birth attendance at delivery (31.7 %) and facility based delivery (26.9 %) were observed among South Asian and Southeast Asian countries [4, 5].

Primary health care is the keystone of a rational health services system in any country [6]. Nearly two-thirds of people in Bangladesh live in rural areas, the majority of which under low socioeconomic conditions [7]. To extend PHC at the grass roots level, including the remotest and hardest to reach areas, the government of the People’s Republic of Bangladesh planned to establish 18,000 community clinics (CCs) over the 5 year period of 1996–2001. Ten thousand seven hundred twenty-three CCs were constructed, out of which 8000 began functioning in the 3 years spanning 1998–2001 [8]. However, after a change in the cabinet of the Government of Bangladesh in 2001, all of the CCs were gradually closed and remained non-functioning for the next 8 years [9, 10]. These CCs started to function again in 2009 under the project entitled, “Revitalization of Community Health Care Initiatives in Bangladesh” and have continued operating since then.

On average, each CC served about 6000 people in its jurisdiction at the time the current survey was conducted. All of the CCs were built up in a public private partnership (PPP) style effort. To establish a CC in a locality, community members donated a piece of land on which the government built the office structure for the CC. The government provided logistic support, including human resources and medicine to start the CC, and maintained this support after the start of operations. The management body of a CC consisted of local community leaders as well as representatives from the government. Functions of CCs included health promotion by providing basic education on health, nutrition, and family planning as well as to providing primary management of medical conditions. In addition, they served as a referral link for emergency and complicated cases [8].

In Bangladesh, Health and Population Sector Program (1998–2003) brought some important changes in the health services including sector wide reforms with the aim of achieving a cost effective service delivery system, especially for poor people, women and children, who commonly suffer more compared to people of other demographics. A village level facility such as the CC thus became demanding to provide Essential Services Package (ESP) at least to these most vulnerable groups of people. Based on this concept, CC system was developed in Bangladesh. However, little information is available yet to testify/describe that the CC system is on the right track to fulfill its purposes, as there was a period of 8 long years of non-functioning, prior to resumption of operations in 2009 [11]. One way to evaluate the usefulness of the CC system could be by assessing the awareness of the community people about CCs as well as by collecting information about their CC visits.

It is essential to address health outcomes based on social and economic background [12]. Health seeking behavior is one of the direct pathways through which socioeconomic status can influence health outcomes [13]. Access to healthcare is very important, and interventions that improve this access should improve the overall health outcomes [14]. However, it is important for rural women of any age, who are lagging behind to their male counterparts, to have adequate knowledge and awareness about community health and healthcare system [15, 16]. Women of child bearing age play vital role in home management in rural Bangladesh, including taking care of children and of elderly people. The data we used in the current study was secondary data from a nationwide survey that sampled women of age 12–49 years.

Therefore, the purpose of this study was to evaluate the associations of socioeconomic determinants of women aged 12–49 years with their awareness about CC, as well as with their CC visits.

## Methods

The required information for the current study was based on secondary data provided by the Bangladesh Demographic and Health Survey-2011 (BDHS-2011). BDHS-2011 was conducted by the National Institute of Population Research and Training (NIPORT) of Bangladesh. Mitra and Associates, a Bangladesh-based research firm, conducted the survey. ICF International of Calverton, Maryland, USA, provided technical assistance for the survey as a part of its international Demographic and Health Survey program (MEASURE DHS) and the U.S. Agency for International Development (USAID) provided financial support to complete the survey. The BDHS-2011 database is available online and can be retrieved through registration. We retrieved and utilized this data with permission for study from the DHS program [17].

BDHS-2011 was a nationwide population survey. The sampling technique used by the BDHS-2011 consisted of a sampling frame adapted from the population census of Bangladesh. The country was divided into seven divisions, which were subdivided into zilas, and each zila was further divided into upazilas. In rural areas, an upazila is divided into union parishads and mouzas; while in urban areas it is divided into wards, which are further divided into mohallas. The sampling frame consisted of enumeration areas (EAs) which were a collection of households in a mouza or in a mohalla. A two-stage cluster sampling was used to collect the data. In the first stage, EAs were selected with probability proportional to the EAs size. In the second stage, households were selected systematically from the selected clusters. The women population of BDHS-2011 survey consisted of 17,842 ever-married women aged 12–49 years from 17,141 households. We included all these 17,842 ever-married women into the current study [18]. BDHS-2011 survey used questions to evaluate awareness, visitation, and services received from the community clinics (CCs). In this survey, the women were asked “Are you aware of any community clinic in your area?” Those who were aware of CCs, they were further asked “Did you visit the community clinic in the past three months?” Women who had visited were further asked “What services did you receive?”.

Dependent variables of our study were (1) the awareness of women regarding CCs, (2) CC visits. In addition, types of services provided by the clinic were also considered as dependent. Explanatory variables were background information including age, residing areas, level of education, number of children, and wealth index.

Descriptive statistics were used to calculate median, and proportions. Bivariate logistic regression was used to calculate odds ratio (OR) and 95 % confidence interval (CI) to examine the associations between awareness and background characteristics. Association between visitation of the CCs and background information was also evaluated. The Statistical Package for Social Science (SPSS) version 21.0 (SPSS Inc., Chicago, IL, USA) was used for all analyses. All tests were two-sided, and statistical significance was considered at P < 0.05.

This study was conducted using secondary data from the DHS. The DHS Program strictly maintains the privacy of participants during data collection. Ethical approval was received both from the ICF International Review Board (IRB) and from the host country IRB before data collection. Detailed information about the study design, data collection procedure, ethical review, informed consent taking, privacy and confidentiality during data collection and data processing is described well somewhere else [18, 19].

## Results

### Background information

The majority of women (38.4 %) belonged to age group 20–29 years (Table 1) with the median age of 30 years. About 36.0 % women completed secondary level of education (Table 1). Nearly half of the women had no children when this survey was conducted, and 23.5 % of the participants came from richest households (Table 1).

**Table 1:** Characteristics of respondents from DHS-2011, Bangladesh (N = 17,842)

Characteristics	Rural		Urban		Total	
	n	%	n	%	n	%
Age (years)						
12–19	1393	12.0	611	9.9	2004	11.2
20–29	4471	38.4	2372	38.3	6843	38.4
30–39	3212	27.6	1778	28.7	4990	28.0
40–49	2570	22.1	1435	23.2	4005	22.4
Education						
No education	3451	29.6	1188	19.2	4639	26.0
Primary	3721	32.0	1611	26.0	5332	29.9
Secondary	3952	33.9	2454	39.6	6406	35.9
Higher secondary	522	4.5	943	15.2	1465	8.2
Number of children						
0	5536	47.5	3230	52.1	8766	49.1
1	4300	36.9	2308	37.2	6608	37.0
≥2	1810	15.5	658	10.6	2468	13.8
Wealth index						
Poorest	2636	22.6	460	7.4	3096	17.4
Poorer	2903	24.9	442	7.1	3345	18.7
Middle	2766	23.8	662	10.7	3428	19.2
Richer	2215	19.0	1562	25.2	3777	21.2
Richest	1126	9.7	3070	49.5	4196	23.5

*DHS* Demographic and Health Survey

### Awareness and services received by women

Approximately 18 % of participating women were aware of CCs; however, only about 17% of them were visiting CCs. Two hundred and four women (6.5 %) received medicine in sickness for their children, 176 (5.6 %) received family planning services, and 80 (2.5 %) received immunization for them and for their children from these CCs.

### Association between awareness of community clinic and background characteristics

Binary logistic regression analysis was performed to examine the association between the awareness of CCs and the participants’ background characteristics (Table 2). Significant association (P < 0.05) was observed between the CC awareness of participants and different strata of their age, their education level, their residence status, their children number, and their wealth index after mutually controlling the potential confounders (age, education, residence, number of children, and wealth index). Women aged 20–29 years showed significantly higher odds of awareness about CCs compared to women aged 12–19 years (adjusted OR = 1.18, 95 % CI 1.03–1.35, P = 0.015). Similarly women who completed primary or secondary level of education showed significantly higher odds of awareness about CCs compared to women who did not go to school (adjusted OR = 1.20, 95 % CI 1.08–1.34, P = 0.001 for primary level educated women; and adjusted OR = 1.35, 95 % CI 1.20–1.52, P < 0.001 for secondary level educated women). Women living in rural areas showed significantly higher odds of awareness about CCs compared to women who lived in urban areas (adjusted OR = 2.72, 95 % CI 2.44–3.04, P < 0.001). Similar significant results were found regarding the wealth index of participants with the higher adjusted OR in poor women compared to women who came from richest households.

**Table 2:** Odds ratios of characteristics towards community clinics awareness of respondents from DHS-2011, Bangladesh (N = 17,842)

Characteristics	Awareness, n (%)	Crude			Adjusted^b^		
		OR	95 % CI	*P*-value^a^	OR	95 % CI	*P*-value
Age (years)							
12–19	351 (11.1)	1	Reference		1	Reference	
20–29	1268 (40.1)	1.07	0.94–1.22	0.301	1.18	1.03–1.35	0.015
30–39	867 (27.4)	0.99	0.86–1.14	0.889	1.15	0.99–1.32	0.066
40–49	678 (21.4)	0.96	0.83–1.11	0.570	1.15	0.98–1.34	0.086
Education							
No education	786 (24.8)	1	Reference		1	Reference	
Primary	1002 (31.7)	1.13	1.02–1.26	0.016	1.20	1.08–1.34	0.001
Secondary	1191 (37.8)	1.13	1.02–1.24	0.020	1.35	1.20–1.52	<0.001
Higher secondary	180 (5.7)	0.69	0.58–0.82	<0.001	1.12	0.92–1.36	0.265
Residence							
Urban	560 (17.7)	1	Reference		1	Reference	
Rural	2604 (82.3)	2.90	2.63–3.19	<0.001	2.72	2.44–3.04	<0.001
No. of children							
0	1543 (48.8)	1.05	0.93–1.18	0.414	1.18	1.04–1.34	0.009
1	1204 (38.1)	1.10	0.97–1.24	0.143	1.18	1.04–1.33	0.011
≥2	417 (3.2)	1	Reference		1	Reference	
Wealth index							
Poorest	607 (19.2)	1.86	1.64–2.12	<0.001	1.21	1.03–1.42	0.018
Poorer	717 (22.7)	2.08	1.84–2.36	<0.001	1.28	1.10–1.49	0.001
Middle	717 (22.7)	2.02	1.79–2.29	<0.001	1.26	1.09–1.45	0.002
Richer	637 (20.1)	1.55	1.36–1.76	<0.001	1.14	1.00–1.31	0.058
Richest	486 (15.4)	1	Reference		1	Reference	

*OR* odds ratio, *CI* confidence interval, *DHS* Demographic and Health Survey

^a^ *P*-value from Wald statistics

^b^Adjusted mutually for all the variables listed in the Table

### Association between CCs visitation and their background characteristics

Significant association (P < 0.05) was observed between the CC visitation by the participants among those who had awareness and different strata of their age, their education level, their number of children and their wealth index except their residence status, (Table 3) after mutually controlling the potential confounders (age, education, residence, number of children, and wealth index). The CC visitation of women aged 20–29 years and aged 30–39 years showed significantly higher odds (Table 3) compared to the CC visitation of women aged 12–19 years (adjusted OR = 1.49, 95 % CI = 1.05–2.11, P = 0.024 for women aged 20–29 years; and adjusted OR = 1.62, 95 % CI = 1.11–2.35, P = 0.012 for women aged 30–39 years). Similarly the CC visitation of women who completed primary level of education showed significantly higher odds (Table 3) compared to the CC visitation of women who did not go to school (adjusted OR = 1.37, 95 % CI = 1.05–1.78, P = 0.019. The CC visitation of women who had no children showed significantly lower odds (Table 3) compared to the CC visitation of women who had two or more children (adjusted OR = 0.68, 95 % CI = 0.50–0.91, P = 0.009). The CC visitation of women who came from poor and middle class families showed significantly higher odds (Table 3) compared to the CC visitation of women who came from rich families (adjusted OR = 2.36, 95 % CI = 1.56–3.55, P < 0.001 for the poorest women; adjusted OR = 2.15, 95 % CI 1.45–3.19, P < 0.001 for poorer women, and adjusted OR = 2.06, 95 % CI = 1.40–3.01, P < 0.001 for women of middle class). No significant association was noted between the CC visitation of women and their residence (Table 3).

**Table 3:** Odds ratios of characteristics towards visitation of community clinics of respondents from DHS-2011. Bangladesh (N = 3162)

Characteristics	Visitation	Crude			Adjusted^4^		
	n (%)	OR^1^	95% CI^2^	P-value^3^	OR	95% CI	P-value
Age (years)							
12-19	46 (8.8)	1	Reference		1	Reference	
20-29	234 (44.8)	1.50	1.07-2.11	0.019	1.49	1.05-2.11	0.024
30-39	153 (29.3)	1.42	0.99-2.03	0.052	1.62	1.11-2.35	0.012
40-49	89 (17.0)	1.00	0.68-1.47	0.992	1.21	0.80-1.84	0.358
Education							
No education	123 (23.6)	1	Reference		1	Reference	
Primary	193 (37.0)	1.28	1.00-1.65	0.049	1.37	1.05-1.78	0.019
Secondary	181 (34.7)	0.96	0.75-1.23	0.747	1.25	0.92-1.69	0.152
Higher	25 (4.8)	0.87	0.55-1.40	0.569	1.56	0.92-2.64	0.098
secondary Residence							
Urban	66 (12.6)	1	Reference		1	Reference	
Rural	456 (87.4)	1.59	1.20-2.09	0.001	1.30	0.97-1.74	0.075
No. of children							
0	224 (42.9)	0.65	0.49-0.85	0.002	0.68	0.50-0.91	0.009
1	211 (40.4)	0.81	0.61-1.07	0.132	0.79	0.59-1.05	0.104
≥2	87 (16.7)	1	Reference		1	Reference	
Wealth index							
Poorest	125 (23.9)	2.31	1.62-3.29	<0.001	2.36	1.56-3.55	<0.001
Poorer	136 (26.1)	2.08	1.47-2.95	<0.001	2.15	1.45-3.19	<0.001
Middle	132 (25.3)	2.01	1.42-2.86	<0.001	2.06	1.40-3.01	<0.001
Richer	80 (15.3)	2.28	0.88-1.86	0.202	1.30	0.88-1.93	0.181
Richest	49 (9.4)	1	Reference		1	Reference	

*DHS* demographic and health survey

^1^ *OR* odds ratio

^2^ *CI* confidence interval

^3^ *P*-value from Wald statistics

^4^Adjusted mutually for all the variables listed in the Table

## Discussion

Low prevalence of awareness and visitation of CCs were identified in the current study among women in Bangladesh which is congruent with the findings of studies on care seeking behaviors and healthcare avoidance that were conducted in the USA [20, 21], Nepal [22] and Nigeria [23]. In the USA, one study [21] reported that only around 21 % of the sampled population visited the emergency department at least once. On the other hand, another study [20] described that about 34 % of the respondents avoided seeing healthcare providers when something wrong was suspected with their health. In Nepal, it was reported in a study that approximately 89 % of women did not seek treatment for their ill children [22], whereas in Nigeria [23], this rate was much higher (93 %). In India only 12.7 % mothers reportedly registered their name at PHC centers [24]. There may be various reasons behind this high prevalence rate of untreated sick children in developing countries; however, lack of easy access to healthcare facilities as well as lack of basic health education system could be major barriers to ensuring primary healthcare for vulnerable people, including children. As the CCs provide easy access for patients because of their convenient location, as well as one of their primary functions being basic health education, CCs could easily play vital roles in ensuring primary healthcare in their respective localities. The establishment cost burden of a CC for the respective government is minimized by sharing the cost with the local community under PPP style agreements, facilitating the quick spread of CCs even in a less developed country like Bangladesh. Such evidence is important for other countries, especially for developing countries, where many people still have limited access to primary healthcare facilities.

Noted low prevalence of awareness as well as of low CC visitation among participants of the current study may indicate lesser efficacy of CCs in Bangladesh. However, it is too early to conclude about efficacy of CCs in Bangladesh because alternative forms of similar services, specifically for women, also exist, such as door-to-door health and family planning services provided by different field level health workers (Health Assistants, Family Welfare Assistants). Primary level health care is also provided from the Union Sub-centers and Upzilla Health Complexes. However, as these centers are not located in each community, not all people have easy access to these centers. CCs on the other hand are very convenient by their location, although other facilities at CCs should be improved to accomplish the goals of their establishment. However, it could be relevant to compare with those of USA, Nepal and Nigeria in the sense that future progress of awareness and visitations could be solely attributed to the impact of CCs, considering current rates as baseline information.

The low rate of health awareness and treatment-seeking behavior in this country could be due to a lack of knowledge about the causation and characteristics of illness, stigma, and lack of privacy. Cultural and religious views may also be striking factors behind such behavior in Bangladesh. In addition, attitudes toward health awareness and treatment-seeking behavior are highly dependent on the degree of motivation to seek appropriate health care. Most people in Bangladesh have fairly low expectations of public health services [9] and largely rely on private services [25]. To improve access to PHC, it is necessary to involve community leaders more actively in health intervention programs that focus on improving attitudes towards health and health care, improve patient service-providers relationships, and the implementation of special beneficiary packages like a voucher scheme for pregnant women [26, 27].

Reports are available to demonstrate the association between age and utilization of medical services. The results of our study indicated that older women were more likely to seek treatment from CCs than younger women (≤19 years). Similar findings were identified among mothers seeking treatment from doctors, nurses, and midwives for delivery complications in Bangladesh [4]. The reason behind this could be the leading position of older mothers at family setting and they have more decision-making power. In addition, older women may be able to gather more information regarding health services, which may have a positive impact on utilization [4]. Since most of the women in Bangladesh bear their children between 20 and 39 years of age, they are naturally more conscious about sickness related to children’s health. As a result, when their children become sick, they utilize the maximum level of available health care services from nearby health facilities. The opposite kind of findings are seen in a study conducted in Nigeria which described younger mothers with better care seeking behaviors than older mothers [23]. This might be due to a cultural issue, wherein young women in Bangladesh might have less decision taking power in their families [28].

Women having primary education were more likely to have awareness and seek treatment from CCs than those who did not go to school. Similar findings were observed in several published studies of various kinds of health care utilization [4, 5, 29–34]. Educated women probably had better access to health information and thus were able to utilize such information optimally. Similarly, they are better equipped for initiating and controlling decision-making affairs regarding health issues [35]. Education is considered one of most dominant socioeconomic determinants of health and health related behaviors. It reflects socio-cultural characteristics of individuals as well [36]. In addition, highly educated mothers are known to be better users of health information and services [37] and are thus expected to have better healthcare seeking behaviors for their ill children. Community groups should be used as a platform to provide health education in addition to using mass media for utmost utilization of PHC.

Participants residing in rural areas were more likely to have awareness compared to their urban counterparts at CC setting. This could be due to better accessibility to CCs among rural women in Bangladesh. CCs are located very near to residential areas, as they are established with the aim to improve the accessibility of PHC, particularly among women and children.

Women who had less than two children they were more likely to have awareness than those who had two or more children, similar to the finding of a study conducted in Nigeria [23]. This might be due to a huge effort by the family planning department having a positive effect on the one or two children strategy in Bangladesh.

Women living with lower wealth index were more likely to have awareness about CCs and they visited CCs more than women of higher wealth index. The reason behind this could be due to limited health services available in CC. There is no certified medical doctor appointed for providing health services in the facility. Only para-medical or trained health care providers provide PHC services in the CCs. Perhaps as the women of higher wealth index had scope to visit graduate medical doctors and more equipped medical centers, they did not visit CCs much.

The strength of the present study is that it used the data from a nationwide demographic and health survey, and then used the cluster sampling method to make our study general population representative. However, several limitations of the current study should be considered. Firstly, we included only female respondents; factors among males may have been different. Secondly, our study design was cross-sectional, which cannot establish a cause and effect relationship. Further cohort studies are needed to establish this relationship. Thirdly, certain missing population groups, such as homeless women, and lack of social and cultural influence may affect heath care seeking behavior from CCs. In addition, our study was conducted only 2 years after the restart of CCs following a long 8-year nonfunctioning period. The prevalence of awareness about CCs and treatment-seeking behavior at CCs might have changed over time. Despite these limitations, we believe that the present findings are important; this was a preliminary study carried out among women aged 12–49 years in Bangladesh, evaluating the associations of socioeconomic determinants with awareness and visitation and is, to our knowledge, the first study addressing the influence of socio-economic determinants on CCs in Bangladesh using nationally representative data.

## Conclusions

Low prevalence of CC awareness and low prevalence of CC visitation were observed among women aged 12–49 years in Bangladesh. Older age, primary education, and lower economic condition were positively associated with CC awareness and visitation of CCs in Bangladesh. Community activities involving healthcare workers and community leaders could play vital roles in increasing awareness and improving access of healthcare services through CCs. The government should provide adequate and appropriate logistics, including human resources and equipment, to ease access to PHC services from CCs.
